# Chronic SIRT1 supplementation in diabetic mice improves endothelial function by suppressing oxidative stress

**DOI:** 10.1093/cvr/cvad102

**Published:** 2023-07-04

**Authors:** Kangmin Yang, Srividya Velagapudi, Alexander Akhmedov, Simon Kraler, Tetiana Lapikova-Bryhinska, Martin O Schmiady, Xiaoping Wu, Leiluo Geng, Giovanni G Camici, Aimin Xu, Thomas F Lüscher

**Affiliations:** Center for Molecular Cardiology, University of Zürich, Wagistrasse 12, 8952 Schlieren, Switzerland; Center for Molecular Cardiology, University of Zürich, Wagistrasse 12, 8952 Schlieren, Switzerland; Center for Molecular Cardiology, University of Zürich, Wagistrasse 12, 8952 Schlieren, Switzerland; Center for Molecular Cardiology, University of Zürich, Wagistrasse 12, 8952 Schlieren, Switzerland; Center for Molecular Cardiology, University of Zürich, Wagistrasse 12, 8952 Schlieren, Switzerland; Department of Cardiac Surgery, University Heart Center, University Hospital Zurich, Rämistrasse 100, 8091 Zurich, Switzerland; State Key Laboratory of Pharmaceutical Biotechnology, Department of Medicine and Department of Pharmacology and Pharmacy, The University of Hong Kong, Sassoon Road 21, Pok Fu Lam, 000000 Hong Kong, China; State Key Laboratory of Pharmaceutical Biotechnology, Department of Medicine and Department of Pharmacology and Pharmacy, The University of Hong Kong, Sassoon Road 21, Pok Fu Lam, 000000 Hong Kong, China; Center for Molecular Cardiology, University of Zürich, Wagistrasse 12, 8952 Schlieren, Switzerland; Department of Research and Education, University Hospital Zurich, Rämistrasse 100, 8091 Zurich, Switzerland; State Key Laboratory of Pharmaceutical Biotechnology, Department of Medicine and Department of Pharmacology and Pharmacy, The University of Hong Kong, Sassoon Road 21, Pok Fu Lam, 000000 Hong Kong, China; Center for Molecular Cardiology, University of Zürich, Wagistrasse 12, 8952 Schlieren, Switzerland

**Keywords:** Sirtuin, Endothelial function, Nitric oxide, Reactive oxidative species, NOX1–NOX4

## Abstract

**Aims:**

Enhancing SIRT1 activity exerts beneficial cardiovascular effects. In diabetes, plasma SIRT1 levels are reduced. We aimed to investigate the therapeutic potential of chronic recombinant murine SIRT1 (rmSIRT1) supplementation to alleviate endothelial and vascular dysfunction in diabetic mice (db/db).

**Methods and results:**

Left internal mammary arteries obtained from patients undergoing coronary artery bypass grafting with or without a diagnosis of diabetes were assayed for SIRT1 protein levels. Twelve-week-old male db/db mice and db/+ controls were treated with vehicle or rmSIRT1 intraperitoneally for 4 weeks, after which carotid artery pulse wave velocity (PWV) and energy expenditure/activity were assessed by ultrasound and metabolic cages, respectively. Aorta, carotid, and mesenteric arteries were isolated to determine endothelial and vascular function using the myograph system.

Arteries obtained from diabetic patients had significantly lower levels of SIRT1 relative to non-diabetics. In line, aortic SIRT1 levels were reduced in db/db mice compared to db/+ mice, while rmSIRT1 supplementation restored SIRT1 levels. Mice receiving rmSIRT1 supplementation displayed increased physical activity and improved vascular compliance as reflected by reduced PWV and attenuated collagen deposition. Aorta of rmSIRT1-treated mice exhibited increased endothelial nitric oxide (eNOS) activity, while endothelium-dependent contractions of their carotid arteries were significantly decreased, with mesenteric resistance arteries showing preserved hyperpolarization. *Ex vivo* incubation with reactive oxygen species (ROS) scavenger Tiron and NADPH oxidase inhibitor apocynin revealed that rmSIRT1 leads to preserved vascular function by suppressing NADPH oxidase (NOX)-related ROS synthesis. Chronic rmSIRT1 treatment resulted in reduced expression of both NOX1 and NOX4, in line with a reduction in aortic protein carbonylation and plasma nitrotyrosine levels.

**Conclusions:**

In diabetic conditions, arterial SIRT1 levels are significantly reduced. Chronic rmSIRT1 supplementation improves endothelial function and vascular compliance by enhancing eNOS activity and suppressing NOX-related oxidative stress. Thus, SIRT1 supplementation may represent novel therapeutic strategy to prevent diabetic vascular disease.

## Graphical Abstract

**Graphical Abstract:**
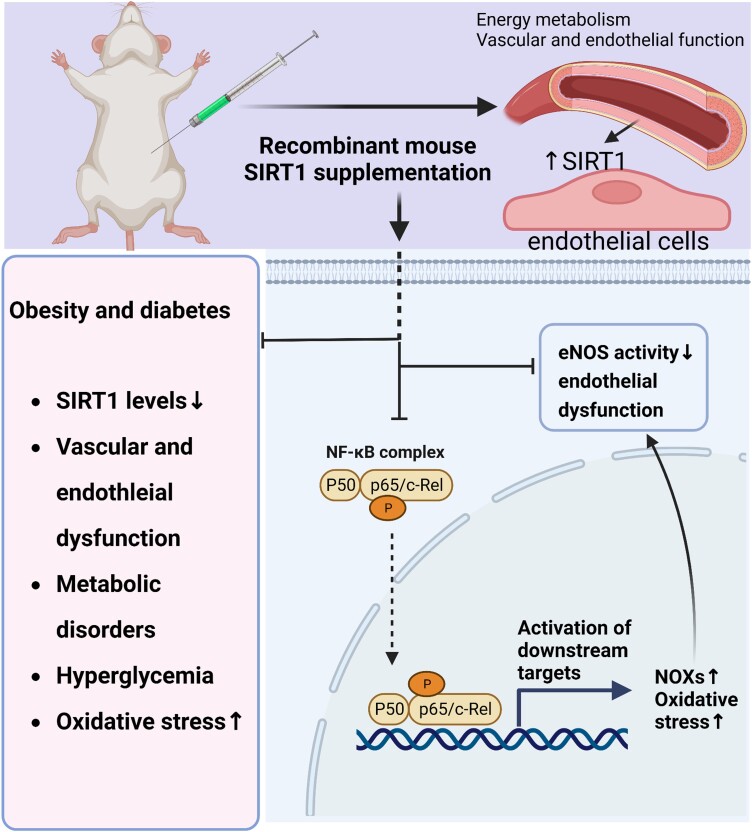

**Time of primary review: 29 days**

## 1. Introduction

Sirtuins and their isoforms SIRT1–SIRT7 are a family of deacetylases exerting protective effects in different organs and promoting longevity in many species. The most extensively studied member of this family is SIRT1, a nicotinamide adenine dinucleotide (NAD^+^)-dependent enzyme. Upon activation by caloric restriction and/or physical exercise, SIRT1 mediates beneficial effects in many organs.^1–3^ The molecular and cellular mechanisms underlying the protective role of endogenous SIRT1 against a variety of age-related-diseases have been studied extensively.^1,4,5^ Indeed, SIRT1 is highly expressed in endothelial cells controlling angiogenesis^6^ and exerting vascular protective effects by suppressing oxidative stress, inflammation, and senescence. Further, it promotes regeneration and smooth muscle proliferation via the regulation of endothelial nitric oxide (eNOS) and a number of transcription factors, including FOXOs, NF-κB, STAT3, PGC1α, PPAR-γ, and p53.^7–9^

The prevalence of diabetes is increasing worldwide and 690 million adults are assumed to be affected by 2045.^10^ Vascular complications, including microvascular complications (i.e. kidney diseases, neuropathy, and retinopathy) and macrovascular (i.e. coronary artery disease, heart failure, and premature death), are the leading causes of mortality and morbidity in these patientss.^11–13^ High levels of intracellular glucose are thought to increase the production of reactive oxidative species (ROS) altering critical downstream pathways in vascular cells, particularly endothelial cells. Indeed, endothelial dysfunction is an early predictor and independent marker of cardiovascular (CV) events, occurring as a hallmark of a wide range of CV diseases. Diabetes-induced endothelial dysfunction is a critical and initiating factor in the genesis of diabetic vascular complications.^14^ Unfortunately, the mechanisms of diabetic vascular disease are neither fully understood nor adequately addressed by current therapeutic strategies that are mainlydirected towards optimal glycaemic control.^12^ Hence, novel therapeutic strategies to improve the metabolic status and particularly cardiovascular dysfunction are still an unmet medical need.

SIRT1 is downregulated in diabetic states.^15^ Conversely, activation of SIRT1 signalling by intermediate fasting or through pharmacological activation may represent an effective therapeutic strategy to prevent clinical sequelae of diabetes.^16,17^ Pharmaceutical and nutriceutical strategies targeting SIRT1 have been studied previously; however, with unsatisfying results largely due to limited target specificity and poor bioavailability.^18–21^

More recently, r SIRT1 has been found to be present extracellularly in circulation. Of note, in a cohort study, serum SIRT1 levels were lower in patients with obesity and liver steatosis than in healthy lean controls, and were inversely correlated with steatosis severity and HbA1c levels.^22^ In another study, plasma levels of SIRT1 were also lower in obese than in lean individuals, and particularly negatively associated with epicardial fat thickness and heart rate in obese subjects.^23^ Further, in obese patients, circulating SIRT1 levels rose significantly with a reduction in fat mass.^24^ In a randomized trial, type 2 diabetics exposed to green cardamon had decreased cardiovascular risk factors including HbA1c, insulin, HOMA-IR, and TG levels, possibly due to effects of intervention on increasing serum SIRT1levels.^25^

Thus, we aimed to investigate whether chronic supplementation of SIRT1 would improve endothelial and vascular dysfunction in a mouse model of daibetes.

## 2. Methods

### 2.1 Animals

All animal experimental protocols were approved by the Committee on the Use of Live Animals in Teaching and Research at the University of Hong Kong, who has obtained full accreditation from the Association for Assessment and Accreditation of Laboratory Animal Care International (AAALAC International) and carried out in accordance with guidelines from Directive 2010/63/EU. Db/db and db/+ male mice were purchased from Jackson Laboratory (Stock No: 000642, Bar Harbor, ME, USA) and housed in the Laboratory Animal Unit during experimental period. All mice were kept in a temperature-controlled room (21 ± 1°C) with a 12 h light/dark cycle. They had free access to water and normal chow (Lab Diet 5053; Purina Mills Inc., Richmond, IN, USA).

After acclimatization for at least 1 week, db/db mice were administrated with rmSIRT1 protein or vehicle, and db/+ mice with vehicle at the age of 12 weeks for a total of 4 weeks via intraperitoneal injection every other day with a dose of 5 μg/mouse/day in a total volume of 100 μL solution. The rmSIRT1 protein was purchased from CUSABIO Science (CUSABIO TECHNOLOGY LLC, Wuhan, China) and dissolved in phosphate buffered saline (PBS). At the end of the treatment period, the activities of mice were recorded in Comprehensive Lab Animal Monitoring System (CLAMS, Columbus Instruments, Hague Ave Columbus, OH 43204, USA). The food intake was monitored weekly.

On the experimental day, mice were anaesthetized with an intraperitoneal injection of sodium pentobarbital (70 mg/kg; Ganes Chemicals, Pennsville, NJ, USA). Then, mice were euthanized by exsanguination through cardiac puncture under anaesthesia. The aortae, carotid, and mesenteric arteries were isolated and dissected for further *ex vivo* studies.

### 2.2 Isometric tension measurements

The aortae, carotid, and mesenteric arteries were put in ice-cold modified Krebs-Ringer solution (Krebs buffer) immediately after being collected from the mice for further removal of the surrounding fat and connective tissues. The buffer was prepared with NaCl 120 mM, KCl 4.76 mM, CaCl_2_ 2.5 mM, MgSO_4_ 1.18 mM, NaHCO_3_ 25.0 mM, NaH_2_PO_4_ 1.18 mM, glucose 5.5 mM, and ethylenediaminetetraacetic acid 0.026 mM, and adjusted to pH 7.4. Aortae with or without endothelium, carotid arteries, and mesenteric arteries of the second order were cut into rings (approximately 2 mm in length). The vessel rings were suspended between stainless steel wires of 45 μm (aorta and carotid artery) or 25 μm (mesenteric artery) in diameter in Halpern–Mulvany myograph system (Danish Myo Technology A/S, Denmark). The chambers were pre-filled with warm (maintained at 37°C) Krebs buffer aerated with 95% O_2_/5% CO_2_ before and during the equilibration of the vessel rings, and an analytical system was used to record and analyse the isometric tension (PowerLab 4SP, AD Instruments, Colorado Springs, CO, USA).

The vessel rings were equilibrated for 30 min and undergo different standard stretching protocols to determine the optimal distension of each ring. For aortic and carotid arteries, the rings were processed with active stretch. They were equilibrated under basal tension of (5 mN for aortic artery and 0.5 mN for carotid artery) and then were stretched step by step and exposed to KCl solution (60 mM) until their contraction to KCl reached the maximum. For the mesenteric arteries, rings were equilibrated without extra tension and then put at the optimized tension by the standard normalizing protocol embedded within the PowerLab 4SP. Rings were allowed to equilibrate again for 20 min at optimal distension. The contraction to KCl at the optimal distension was used as the reference contraction of each ring. For the assay of relaxation response, vessels rings were pre-contracted with phenylephrine (10^−7.5^–10^−6^ M) to 60–70% of the maximum contraction to KCl.

The examination of the response of vascular smooth muscle cells was performed after removing endothelium. Endothelial cells were removed by perfusion of the arteries with a Triton solution (0.3 mL; 0.5% in Krebs). The successful removal of the endothelial cells was confirmed by the absence of relaxation to acetylcholine.

### 2.3 Vascular ultrasound

Doppler ultrasound was performed using a Vevo 2100 Imaging System (Fujifilm Visual-Sonics, Canada) to assess the pulse wave velocity (PWV) from the left common carotid artery. Mice were firstly put in an anaesthesia induction chamber filled with 2.5% isoflurane in 1 L/min pure oxygen till unresponsiveness to toe pinch, then was placed on a temperature-controlled board in a supine position supplied with anaesthetic gas flow (1.5% isoflurane). The limbs of the mice were coated with conductive gel and taped on the electrocardiogram electrodes embedded in the board. The heart rate was adjusted and maintained at 400 to 450 beats per minute (bpm) during the whole assessment by adjusting the anaesthetic gas flow. The fur was chemically removed from the cervical region with depilatory cream and coated with acoustic coupling gel (Nair™, Carter-Horner, Mississauga, ON, Canada). Using B-mode imaging, the transducer was positioned to provide a longitudinal section of the mouse carotid artery, with the region of interest located in the focal zone of the transducer. Pulsed-wave Doppler images were acquired immediately after the B-mode ones using the same scan projection.

### 2.4 Energy metabolic cage housing

Metabolic monitoring was performed using a Comprehensive Lab Animal Monitoring System (CLAMS, Columbus Instruments, Columbus, OH, USA) that simultaneously measures whole-body O_2_ consumption, CO_2_ production, and physical movements. Mice were put in the chambers for 2 days for acclimatation before the experiment to minimize the changes in housing environments. Data were collected every 10 min for each mouse over a period of 2 days. Metabolic parameters measured included oxygen consumption (V_O_2__) and CO_2_ production (V_CO_2__), such that respiratory exchange ratio (RER = V_CO_2__/V_O_2__) and rate of energy expenditure could be calculated. Rate of energy expenditure was calculated from V_O_2__ (l/h) and RER {Heat production = ([3.815 + (1.232 ∗ RER)] ∗ V_O_2__)}. The locomotor activity was also measured using two sets of infrared beams lining the cages to monitor linear and vertical movement of the mice. The system was operated with an air intake of 0.6 L/min per chamber and an extracted ouflow of 0.4 L/min. The lighting was set to be on a normal 12 h light/dark cycle (light on from 7 am to 7 pm as the light cycle). All measurements were taken at an ambient temperature of 21–22°C. Metabolic rate and physical activity were averaged for the whole study period with the exceptions of the first five time points that tend to be influenced by animal handling at the beginning of studies. Body weight was used in the normalization of metabolic rate data.

### 2.5 Western blotting

Isolated murine vessel tissues were homogenized in radioimmunoprecipitation assay (RIPA) buffer containing a cocktail of proteinase and phosphatase inhibitors (Pierce Protease Inhibitor Tablets, Thermo Scientific™, MA, USA). The homogenate was cleared by centrifugation (12 000 *g* per minute, for 10 min at 4°C). The protein concentration was determined using Pierce™ BCA protein Assay Kit (Thermo Scientific™, MA, USA). Twenty micrograms of total proteins were subjected to SDS-PAGE followed by blotting to polyvinylidene difluoride membranes. The membranes were then probed overnight at 4°C with primary antibodies (anti-total eNOS, 1:1000 of dilution, Cell Signaling Technology; anti-p^Ser1177^eNOS, 1:500 of dilution, BD Biosciences; anti-NOX1, 1:1000 of dilution, Abcam; anti-NOX2, 1:1000 of dilution, Invitrogen-Thermo Scientific; anti-NOX4, 1:1000 of dilution, Invitrogen-Thermo Scientific; anti-NF-κB p65 (phospho S536), 1:1000 of dilution, Abcam) followed by secondary antibodies [anti-goat IgG, (H + L)-HRP, anti-rabbit IgG, HRP, or anti-mouse IgG, HRP] for 60 min at room temperature before enhanced chemiluminescence detection with Clarity Western ECL Substrate (Bio-Rad, Hercules, CA, USA). The validation of the rmSIRT1 was performed in western blotting using monoclonal anti-SIRT1 (1:1000, Abcam). For detection of carbonylation in aorta, the polyvinylidenedifluoride membranes were firstly derivatized with 2,4-dinitrophenylhydrazine and then processed to blocking with 5% non-fatty milk, followed with primary anti-dinitrophenol (DNP) antibody (1:200, LifeSpan BioSciences, Inc.) overnight at 4°C.

### 2.6 Measurement of SIRT1 levels in arteries

In mice, freshly isolated aortae were cleaned from surrounding fat and connective tissues lysed in RIPA buffer using Soft Tissue Homogenizer (Labgene Scientific) and then stored at −80°C until used for SIRT1 enzyme-linked immunosorbent assay (ELISA) assays according to the instructions of the manufacturer. In patients undergoing coronary artery bypass grafting, the left internal mammary artery (LIMA) was prepared for its use as a bypass graft, and the distal part of the blood vessel was cut, flushed with PBS, and immediately snap-frozen in liquid nitrogen until further processing. Patients with a confirmed diagnosis of type 2 diabetes were matched to non-diabetic individuals using optimal full matching based on sex and age. The LIMA tissues obtained from these patients (*n* = 6–18/group) were lysed in RIPA buffer. Protein concentration was assessed using the Pierce™ BCA Protein Assay Kit (Thermo Scientific™, MA, USA).

Vascular SIRT1 levels were determined in a blinded fashion using a commercially available SIRT1 ELISA (Abcam, Cambridge, UK and LifeSpan BioSciences, Inc.). SIRT1 levels were normalized to total protein concentration. All patients provided informed consent, and the study was approved by the cantonal ethics committee.

### 2.7 Measurement of plasma nitrotyrosine

The level of nitrotyrosine was measured using commercial ELISA kit according to manufacturer’s instructions (ab210603, Abcam, Cambridge, UK).

### 2.8 Pathohistology

Ascending aortae were cut when mice were sacrificed and immediately put in 4% paraformaldehyde for 24 h for fixation. The fixed tissues were sent to professional histology lab (Sophistolab, Zurich, Switzerland) for the following processing and immunohistological/pathohistology staining. The quantification analysis was performed with ImageJ software.

### 2.9 Data and statistical analysis

For data and analysis from the organ chamber experiments, contractions are expressed as the percentages to the response to 60 mM KCl obtained at the beginning of the experiments. Relaxations were calculated as the percentages to the phenylephrine-induced contractions. The results are shown as mean ± S.e.m. or mean ± Sd with *n* referring to the number of animals used. The responses were also analysed and compared with the area under the concentration–response curve (AUC). Concentration–response curve were plotted with Prism version 7 (GraphPad Software, San Diego, CA, USA). Statistical analyses were performed by using Student’s unpaired *t*-test, with or without Welch’s correction, as appropriate, for two group comparisons. To compare values of three groups, a one-way analysis of variance (ANOVA) was used, followed by Tukey *post hoc* test. The intensity of western blot was calculated using the computerized program (ImageJ software, National Institutes of Health). Optimal full matching in the human cohort was achieved using the R package ‘matchit’ (version 4.4.0; R Foundation, Vienna, Austria). Covariate balance was assessed visually and by interrogating the absolute standardized mean difference of included covariates. *P* values less than 0.05 were considered to indicate statistically significant differences.

### 2.10 Materials

Recombinant murine SIRT1 protein was purchased from CUSABIO Science (CUSABIO TECHNOLOGY LLC, Wuhan, China). Anti-total eNOS (Cell Signaling Technology Cat# 32027) and anti-phosphorylated (Ser1177) eNOS antibodies were purchased from BD Biosciences (San Jose, CA, USA); anti-NOX1 and anti-phosphorylated p65 were purchased from Abcam. Anti-NOX2 and NOX4 were purchased from Invitrogen-Thermo Scientific. Anti-DNP were purchased from LifeSpan BioSciences. Anti-rabbit and anti-mouse IgG secondary antibodies were purchased from SouthernBiotech.

## 3. Results

### 3.1 SIRT1 levels in arteries

**Figure 1:**
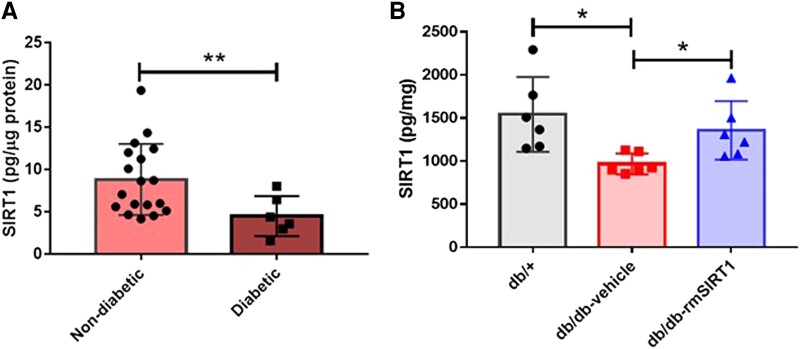
Levels of SIRT1 in arteries and structural difference with or without rmSIRT supplementation. (*A*) LIMA from patients (*n* = 18 patients in non-diabetic group and 6 patients in diabetic group) and (*B*) aorta from three groups of mice were assayed for SIRT1 level by ELISA and normalized to total protein concentration in tissue lysate (*n* = 6 mice in each group). Mean ± SD. ^*^P < 0.05, one-way ANOVA; ^**^P < 0.01, Student’s *t*-test (unpaired).

LIMA tissues obtained from diabetic patients with coronary artery disease displayed lower SIRT1 levels than those from non-diabetic patients [3.99 (2.63–6.84) vs. 7.87 (5.49–12.1), *P* = 0.0064], indicating a strong association of diminished vascular SIRT1 levels with diabetes and diabetic vascular dysfunction (*Figure 1A*).

In line, protein levels of aortic SIRT1 were markedly decreased in *db/db* mice as compared to *db/+* mice (986 ± 120 vs. 1479 ± 464 pg/mg, *P* = 0.026). Following rmSIRT1 supplementation over 4 weeks, SIRT1 levels increased significantly and intriguingly reached similar levels to those observed in lean controls (1357 ± 339 vs. 1479 ± 464 pg/mg, *P* = 0.615; *Figure 1B*).

### 3.2 SIRT1 and energy metabolism

**Figure 2:**
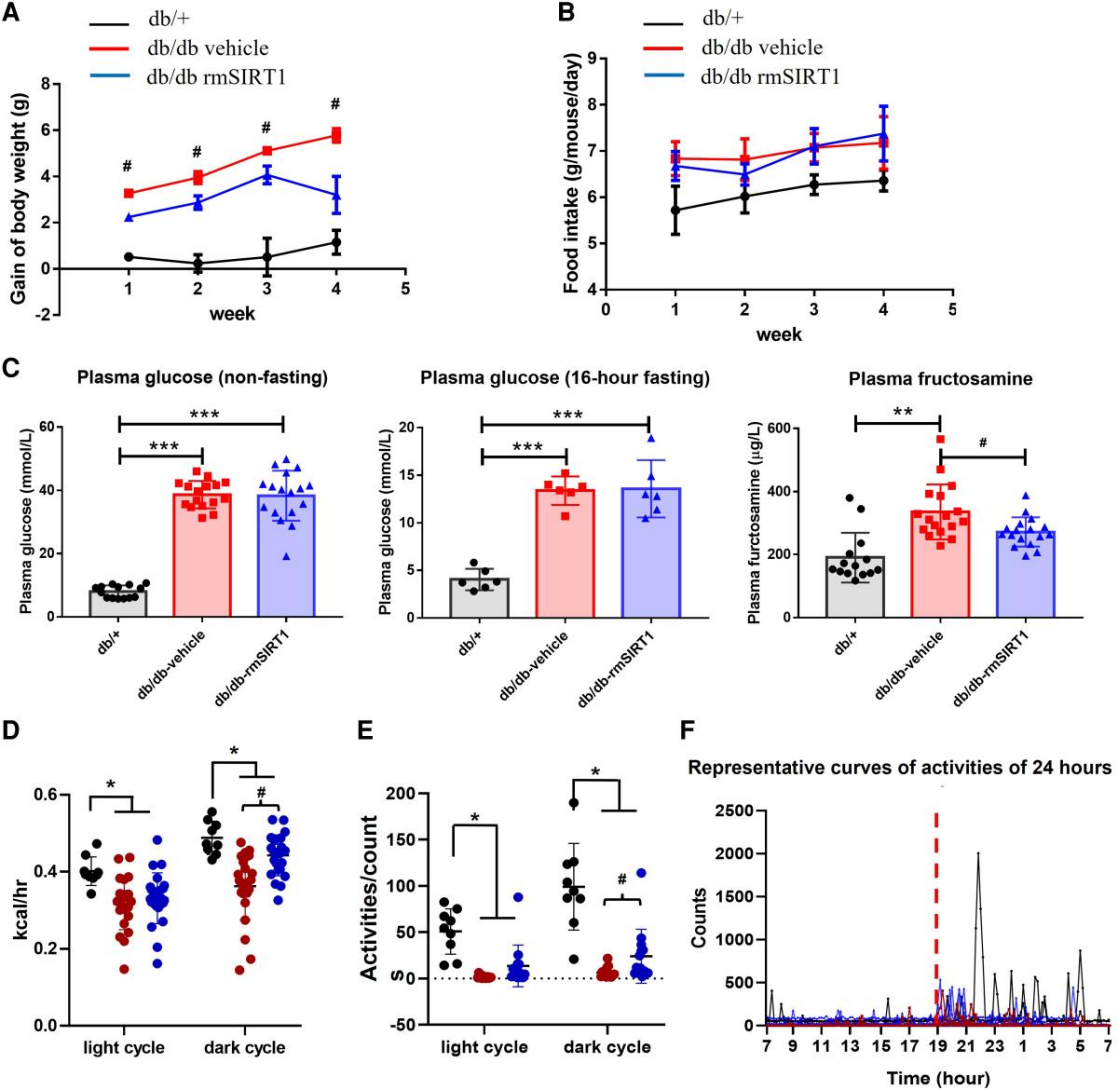
Effects of rmSIRT1 supplementation on metabolic parameters. (*A*) Weekly gain of body weight (*n* = 15 mice in each group); (*B*) food intake (*n* = 15 mice in each group); (*C*) glucose level with and without 16 h fasting and fructosamine level in plasma (*n* = 6–14, 6–17, 6–18 mice in db/+, vehicle, and rmSIRT1 groups, respectively); (*D*) rate of energy expenditure (*n* = 9, 24, and 24 mice in db/+, vehicle, and rmSIRT1 groups); (*E*) physical activity (*n* = 9, 14, and 14 mice in db/+, vehicle, and rmSIRT1 groups); (*F*) representative 24 h physical activity. Mean ± S.E.M. in curves. Compared to db/+ group, ^*^P < 0.05, ^**^P < 0.01, ^***^P < 0.001; compared to vehicle group, ^#^*P* < 0.05. One-way ANOVA.

Chronic rmSIRT1 supplementation significantly alleviated weight gain in db/db mice from 12 weeks up to 16 weeks of age (*Figure 2A*). As food intake remained unaffected by rmSIRT1 treatment (*Figure 2B*), energy expenditure may have changed. Although no significant differences were observed in the fasting and non-fasting glucose levels between treated and non-treated groups, total glycated serum proteins reflected by fructamine levels were significantly down-regulated by chronic rmSIRT1 supplementation, indicating a better long-term handling of glucose level in the treated period (*Figure 2C*). Further, energy expenditure was significantly increased in the dark cycle in diabetic mice treated with rmSIRT1 compared to controls, while there was no statistical difference in the light cycle (*Figure 2D*). Body weight gain was associated with significant changes in different components of energy expenditure, including locomotor activities. Monitoring and analysis of mice by CLAMS also revealed that the locomotor activities were significantly increased among the mice with rmSIRT1 supplement (*Figure 2E, F*).

### 3.3 SIRT1 blunts increased arterial stiffness

**Figure 3:**
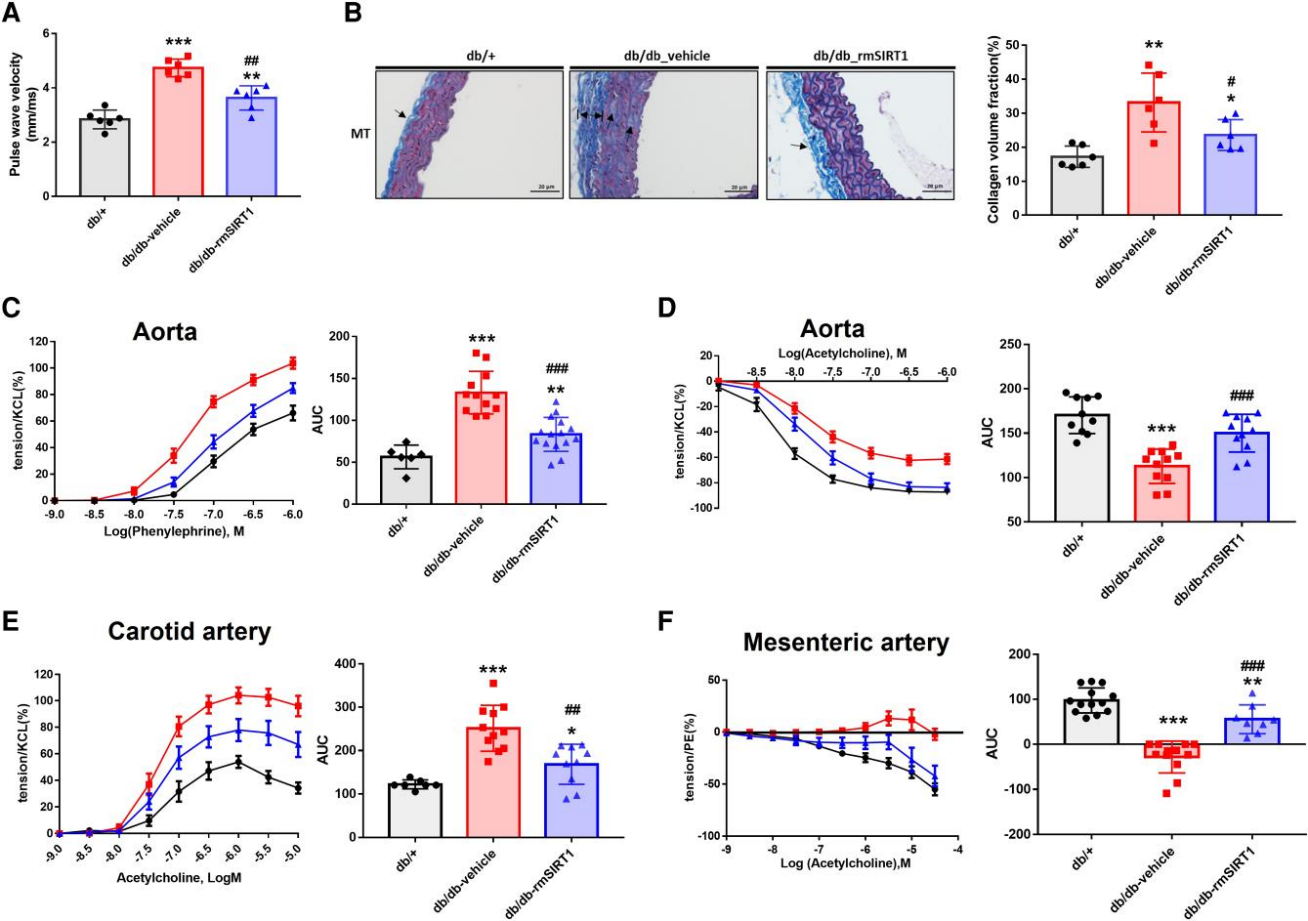
Effects of rmSIRT1 supplementation on vascular structure and function of db/db mice. (*A*) Pulse wave velocity of carotid arteries as assessed by ultrasound (*n* = 6 mice in each group); (*B*) left: representative pictures of Masson’s Trichrome Staining (MT) (*n* = 6 mice in each group). Arrows indicate the thickness of collagen layer in adventitia and collagen deposition in extracellular matrix in media of aorta; right: the collagen volume fraction (%). (*C*) Concentration-dependent contractions to phenylephrine of aorta (*n* = 6, 12, and 15 mice in each group); (*D*) concentration-dependent relaxations to acetylcholine of aorta (*n* = 10, 11, and 11 mice in db/+, vehicle, and rmSIRT1 groups); (*E*) concentration-dependent contractions to acetylcholine of the carotid artery with incubation of eNOS inhibitor (L-NAME 10^−4^ M) (*n* = 7, 11, and 10 mice in db/+, vehicle, and rmSIRT1 groups); (*F*) concentration-dependent endothelium-dependent relaxations to acetylcholine of mesenteric artery with incubation of eNOS inhibitor (L-NAME 10^−4^ M) and COX inhibitor (indomethacin, 3 × 10^−5^ M). Left panel: dose–response curves; right panel: area under curve (*n* = 13, 12, and 8 in db/+, vehicle, and rmSIRT1 groups). Mean ± S.E.M. in curves. Compared to db/+ group, ^*^P < 0.05, ^**^P < 0.01, ^***^P < 0.001; compared to db/db vehicle, ^#^*P* < 0.05, ^##^*P* < 0.01, ^###^*P* < 0.001. One-way ANOVA.

Next, PWV, a proxy of arterial stiffness, was assessed *in vivo*. As expected, diabetic db/db mice exhibited markedly increased PWV as compared to non-diabetic controls. Notably, rmSIRT1 supplementation blunted the initially increased PWV in db/db mice (*Figure 3A*), suggesting reinstated vascular function by rmSIRT1 treatment *in vivo*. At the structural level, diabetic conditions lead to thickening of the adventitial collagen layer with increased collagen deposition in the media, a phenomenon that was attenuated upon rmSIRT1 treatment (*Figure 3B*).

### 3.4 SIRT1 improves vascular dysfunction

In line, *db/db* mice exhibited marked vascular dysfunction of the aorta as reflected by more pronounced contractions to an α-receptor agonist and reduced relaxation to a muscarinic agonist, respectively (*Figure 3C, D*). Chronic treatment with rmSIRT1 significantly reduced contractions of the aorta to increasing concentrations of phenylephrine (10^−9^–10^−6^ M), while relaxations to increasing concentrations of acetylcholine (10^−9^–10^−6^ M) were significantly augmented compared to vehicle-treated animals (*Figure 3C, D*).

In carotid arteries, acetylcholine caused dose-dependent contractions in the presence of an eNOS inhibitor (L-NAME; 10^−4^ M), which were previously shown to be due to endothelial-derived prostanoid vasocontracting factors (EDCF).^26^ Acetylcholine-induced contractions were higher in magnitude in db/db mice than in lean controls. Mice treated with rmSIRT1 had significantly reduced contractions compared to vehicle-treated db/db mice (*Figure 3E*).

To assess the responsiveness attributable to endothelial derived hyperpolarization (EDH),^27^ changes in tension of mesenteric resistance arteries to increasing concentrations of acetylcholine (10^−9^–10^−6^ M) were recorded in the presence of L-NAME (10^−4^ M) and the non-selective cyclooxygenase inhibitor indomethacin (3 × 10^−5^ M). After incubation with L-NAME and indomethacin, acetylcholine still induced dose-dependent relaxations of mesenteric resistance arteries of db/+ mice. However, EDH was abolished in db/db mice resulting in acetylcholine-induced contractions. Chronic treatment with rmSIRT1 significantly improved that response in mesenteric arteries, though not reaching levels seen in lean db/− mice (*Figure 3F*).

### 3.5 SIRT1 recovers eNOS function

**Figure 4:**
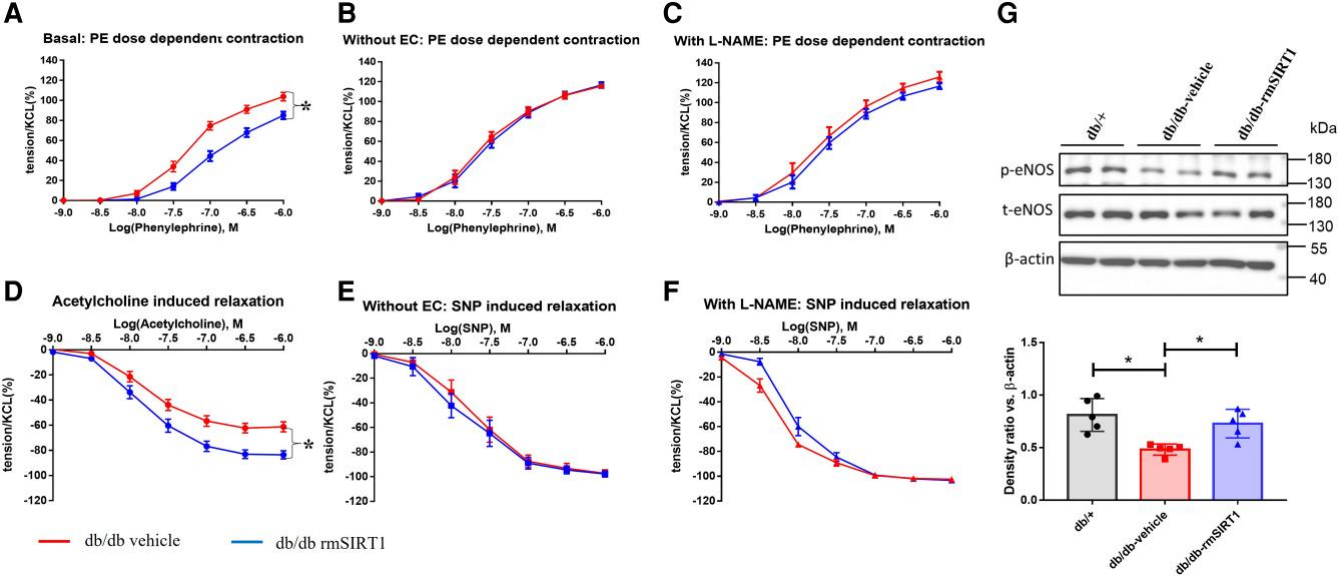
Effects of rmSIRT1 supplementation on aorta is endothelium and eNOS dependent. (*A*) Concentration-dependent contractions to phenylephrine of intact aortic rings (*n* = 12 mice in vehicle group and 15 mice in rmSIRT1 group); (*B*) concentration-dependent contractions to phenylephrine in aortic rings after removal of endothelium (*n* = 7 in each group). (*C*) Concentration-dependent contractions to phenylephrine in aortic rings with pre-incubation of L-NAME (10^−4^ M) (*n* = 7 mice in each group). (*D*) Concentration-dependent relaxations to acetylcholine of intact aortic rings (*n* = 11 mice in each group); (*E*) concentration-dependent relaxations to sodium nitroprusside of aortic rings after removal of endothelium (*n* = 7 mice in each group); (*F*) concentration-dependent relaxations to sodium nitroprusside of aortic rings after pre-incubation of L-NAME (10^−4^ M) (*n* = 7 mice in each group); (*G*) western blotting analysis of eNOS expression (*n* = 5 mice in each group). Mean ± S.E.M. in curves. ^*^P < 0.05 in comparisons, Student’s *t*-test (unpaired) for the comparisons of two groups, and one-way ANOVA if more than two groups were compared.

Next, we aimed to delineate the molecular mechanisms underlying the observed protective effects of rmSIRT1 supplementation on vascular function in diabetic animals. First, we studied whether these effects were attributable to the endothelium rather than vascular smooth muscle cells. To this end, vascular responsiveness to vasoconstrictors and vasodilators with and without endothelium was assessed. Of note, after removal of the endothelium, phenylephrine-induced contractions (10^−10^–10^−6^ M) and sodium nitroprusside-induced relaxations (10^−10^–10^−6^ M) were reaching similar levels in rmSIRT1-treated and untreated *db/db* mice (*Figure 4B, E*), indicating a critical involvement of the endothelium in the protective effects of rmSIRT1 supplementation. To delineate whether or not eNOS was involved, intact aortic rings from both rmSIRT1- and vehicle-injected *db/db* mice were incubated with L-NAME (10^−4^ M) and vascular responses compared between the two groups. Blockage of eNOS abolished the differences observed between the two groups and thus the effects of rmSIRT1 treatment, suggesting eNOS as a key player mediating the protective effects of rmSIRT1 (*Figure 4C, F*).

As eNOS is one of the most important enzymes regulating endothelial function, its activation status was investigated by western blotting. Indeed, the ratio of eNOS phosphorylated at serine 1177 (S1177) (p-eNOS) to total eNOS (t-eNOS) was significantly down-regulated in db/db mice, whereas it was significantly increased after rmSIRT1 treatment (*Figure 4G*).

### 3.6 SIRT1 reduces oxidative stress by inhibiting NADPH oxidase

**Figure 5:**
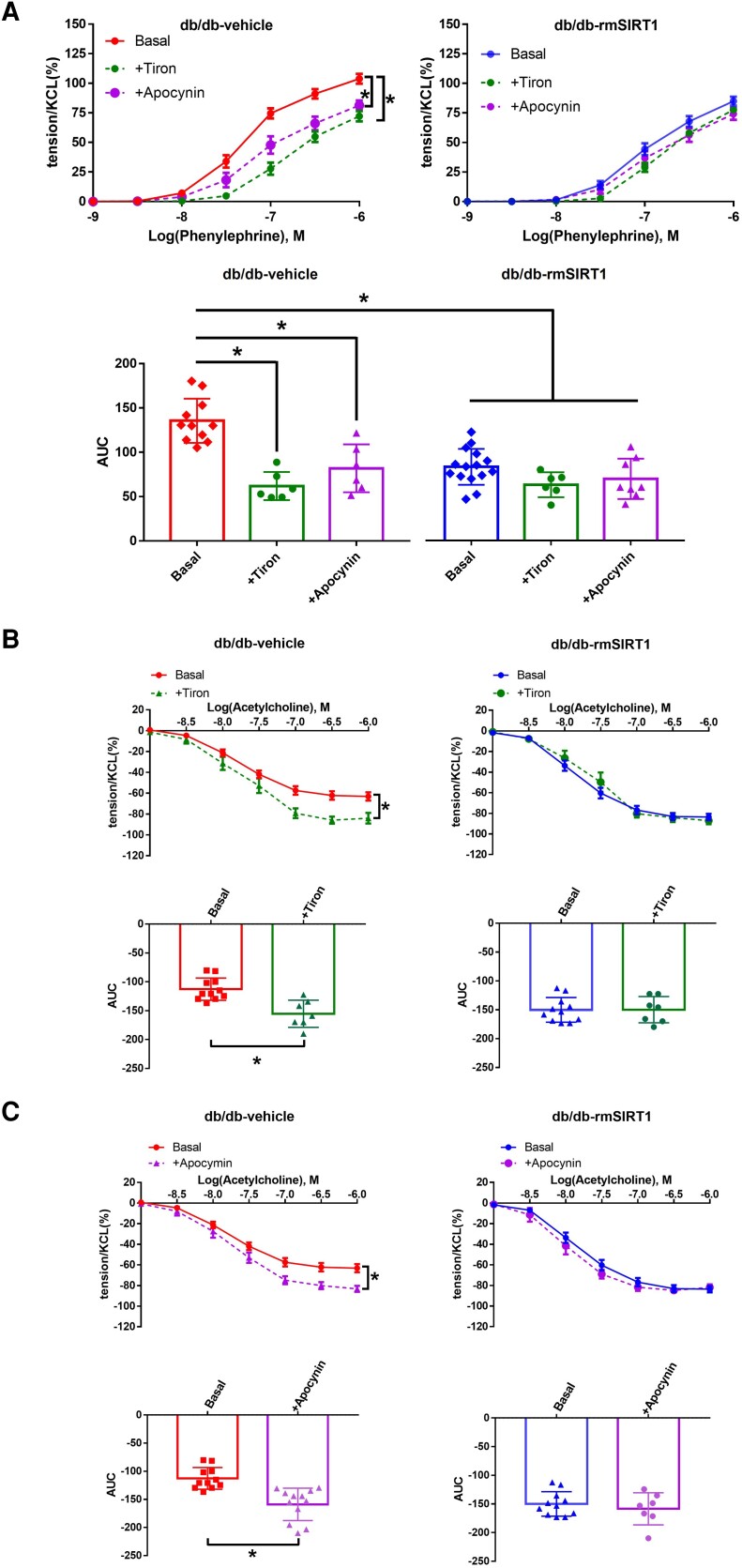
Comparisons of the effects of different inhibitors on aortic function between rmSIRT1-treated and non-treated db/db mice. (*A*) Concentration-dependent contractions to phenylephrine at control conditions, with the ROS scavenger (Tiron 10^−4^ M), or NADPH oxidase inhibitor (apocynin 10^−4^ M) (*n* = 6–11 mice in vehicle group and 6–15 mice in rmSIRT1 group); (*B*) concentration-dependent relaxations to acetylcholine with and without Tiron. The responses were compared between db/db-vehicle (left) and db/db-rmSIRT1 groups (right) (*n* = 7–11 mice in vehicle group and 6–15 mice in rmSIRT1 group); (*C*) concentration-dependent relaxations to acetylcholine with and without apocynin (*n* = 11–12 mice in vehicle group and 7–11 mice in rmSIRT1 group). Upper panel: dose–response curves; lower panel: area under curves. Mean ± S.E.M. in curves, ^*^P < 0.05 vs. basal response of control group, Student’s *t*-test (unpaired) for the comparisons of two groups, and one-way ANOVA if more than two groups were compared.

Increased oxidative stress contributes critically to impaired vascular function during aging and is particularly relevant to vascular dysfunction in diabetics.^28,29^ Here, we observed that incubation of aortic rings with the ROS scavenger Tiron (10^−4^ M) significantly improved vascular function by reducing endothelium-dependent contractions and increasing vasodilation in *db/db* mice supplemented with vehicle (*Figure 5A, B*). However, after chronic *in vivo* treatment with rmSIRT1, the effects of Tiron were abolished to levels observed in the control group, indicating that rmSIRT1 improves vascular function by reducing oxidative stress (*Figure 5A, B*). We then hypothesized that NADPH oxidase may be regulated by rmSIRT1 treatment leading to reduced ROS production either directly or indirectly. To that end, aortic rings were incubated with the non-selective NADPH oxidase inhibitor apocynin (10^−4^ M) prior to the addition of vasoconstrictors or vasodilating agents. In db/db mice receiving chronic supplementation of rmSIRT1, apocynin lost its beneficial vascular effects, indicating that the deteriorating effects of NAPDH oxidases were alleviated by *in vivo* treatment with the protein (*Figure 5A, C*).

**Figure 6:**
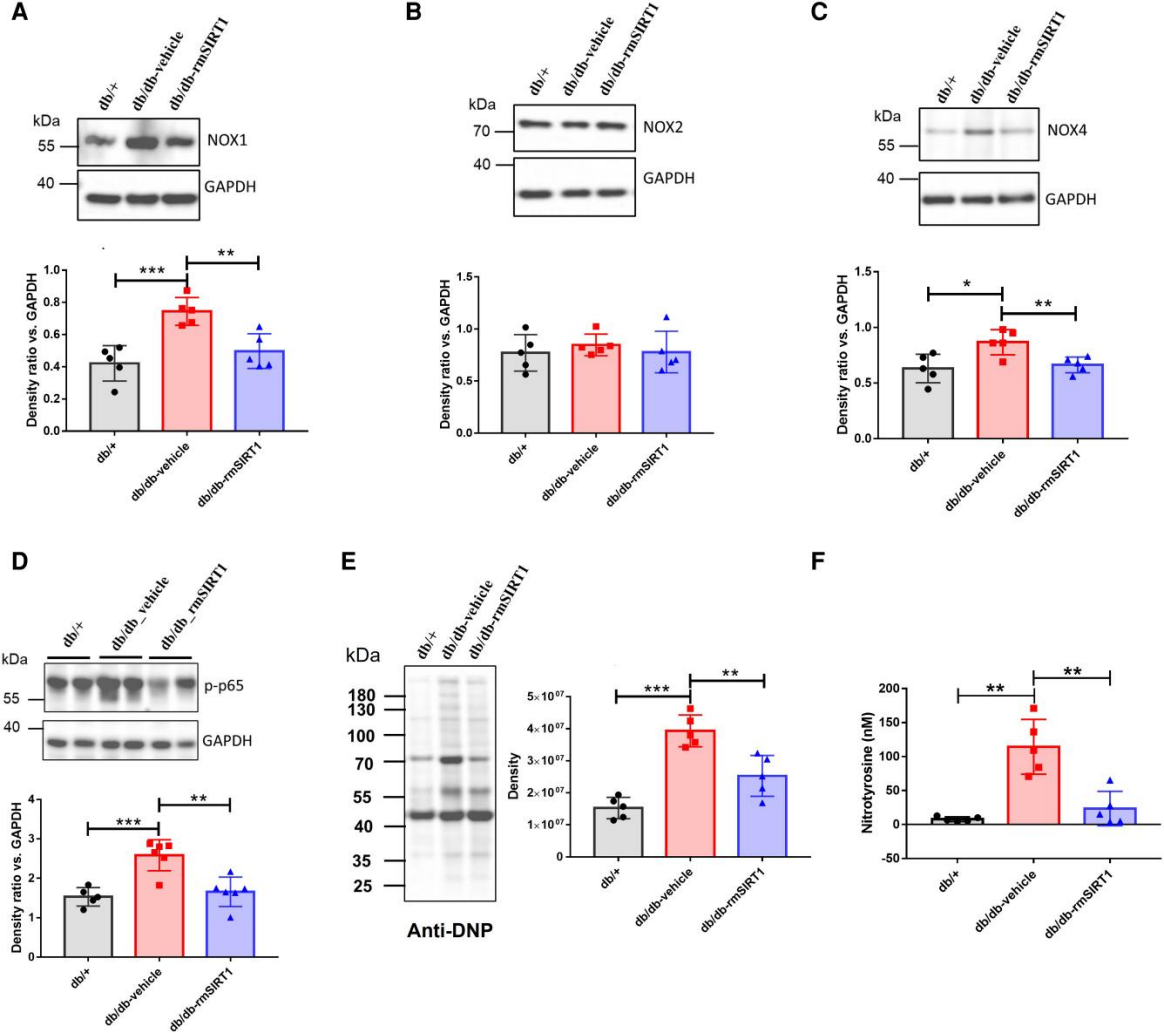
Regulation of protein expressions by rmSIRT supplementation in intact aortas. Western blotting analysis of NOX1 (*A*) (*n* = 5), NOX2 (*B*) (*n* = 5), NOX4 (*C*) (*n* = 5), phosphorylated P65 (S276) (*D*) (*n* = 5–6), and protein carbonylation using anti-dinitrophenyl antibody (anti-DNP) (*E*) (*n* = 5) in the aorta and plasma nitrotyrosine (*F*) (*n* = 5) of control (db/+), vehicle-treated (db/db-vehicle), and rmSIRT1-treated mice (db/db-rmSIRT1). Results were quantified by densitometry and presented as a ratio of density of protein bands vs. those of GAPDH from the same sample. Results of protein carbonylation were quantified by densitometry and presented as density of protein bands from samples with the equal amount of total protein. Data are shown as mean ± SD. Data were analysed as using one-way ANOVA. ^*^P < 0.05, ^**^P < 0.01, ^***^P < 0.001.

To investigate the expression of key proteins mediating the observed changes in vascular function, aortic lysates were used for *western blotting*. NADPH oxidase is a major source of ROS in diabetes. Among different isoforms of NADPH oxidases, NOX1, NOX2, and NOX4 are abundantly expressed in the vasculature. Among them, NOX isoforms 1 and 4 were up-regulated in the aorta of db/db mice, and then were down-regulated upon treatment with rmSIRT1 (*Figure 6A, C*). However, no difference was observed in the expression of NOX2 between the groups (*Figure 6B*). NOX expression and activity can be regulated involving an NF-ĸB-dependent mechanism.^30–32^ Interestingly, at baseline, diabetic mice showed increased NF-ĸB activation, as determined by p65 phosphorylation, but with a marked attenuation following rmSIRT1 treatment, implicating that the reduction of NOXs upon rmSIRT1 supplementation may involve blunted activation of the NF-ĸB pathway (*Figure 6D*).

Protein carbonylation is an established marker of oxidative stress. Following the investigation of the key components involved in ROS production, oxidative stress was assessed by measuring protein carbonylation in aortic tissue. Remarkably, protein carbonylation was increased in aorta of db/db mice and significantly reduced upon chronic treatment of rmSIRT1 (*Figure 6E*). Nitrotyrosine is another well acknowledged surrogate of oxidative stress. While highly increased in diabetic mice, nitrotyrosine levels were remarkedly reduced following rmSIRT1 treatment (*Figure 6F*).

## 4. Discussion

Here, we report that in mice with obesity-induced diabetes, chronic restoration of aortic SIRT1 levels (i) increased night time activity and energy expenditure of the mice, (ii) reduced weight gain, (iii) improved glucose metabolism, (iv) significantly improved endothelial dysfunction, (v) reduced increased PWV, an index of vascular compliance due to reversed vascular remodelling, (vi) restoring eNOS activity by increasing phosphorylation at its active site Ser1177, (vii) down-regulated NOX1 and NOX4, and (viii) eventually attenuated oxidative stress in the vessel wall. We further examined SIRT1 tissue levels in human arteries and found reduced vascular levels of the protein in patients with diabetes. Thus, SIRT1 supplementation represents a novel therapeutic strategy preventing diabetic vascular disease.

High glucose exposure induces endothelial dysfunction and decreases vascular SIRT1 expression, which in turn accelerates functional abnormalities. Based on studies in which endogenous SIRT1 was activated or silenced, it has suggested that the deacetylase may exert a protective role in diabetic vasculopathy.^15,33^ However, to our knowledge, chronic supplementation of the decreased SIRT1 plasma levels in diabetes and their relation to endothelial and vascular dysfunction *in vivo* has not been studied. Here, we, for the first time, demonstrate that in diabetic mice, restoration of the aortic SIRT1 levels indeed improves endothelial and vascular function and structure.

Similar to obese patients with diabetes, *db/db* mice markedly gained body weight during the study period, while *db/db* mice chronically supplemented with rmSIRT1 gained significantly less weight. This is likely related to the fact that after rmSIRT1 supplementation, mice were physically more active particularly during the dark cycle (the most active time period in mice) and thus increased their energy expenditure, while food intake remained unaltered. Similarly, glycaemia of the diabetic mice was significantly alleviated as reflected by reduced levels of fructamine, a well-established marker of total glycated serum proteins that provides a longer time window of glucose control.^34^ However, the effects on blood glucose level were modest and may result from overall improved energy metabolism, although the precise mechanisms remain to be further clarified. Thus, the modest glucose-lowering effects of rmSIRT1 supplementation are unlikely to explain the profound effects of the intervention on endothelial and vascular function and structure.

Remarkably, eNOS activity was improved by rmSIRT1 via increased phosphorylation at its Ser1177 site. Indeed, SIRT1 is known to interact with eNOS expression and activation.^35–38^ In addition, increased physical activity during rmSIRT1 supplementation, as reflected by increased night time activity, might have increased blood flow, and thus shear stress to endothelial cells, an effect that is known to cause eNOS up-regulation via activation of shear stress responsive elements in its promoter.^39–41^

*Db/db* mice spontaneously exhibited pronounced endothelial and vascular dysfunction as reflected by markedly increased arterial stiffness and in turn, increased PWV, enhanced vasoconstriction to phenylephrine, as well as blunted endothelium-dependent relaxations to acetylcholine. Importantly, SIRT1 supplementation improved endothelial function, as reflected by endothelium-dependent blunting of the vasconstriction to phenylephrine and enhanced endothelium-dependent relaxation to acetylcholine. Furthermore, NO- and prostanoid-independent endothelium-dependent hyperpolarization of mesenteric resistance arteries was blunted as well, while contraction to a cyclooxygenase-dependent EDCF was increased. Again, all these vascular alterations were significantly improved by chronic treatment with the rmSIRT1 protein. It is well recognized that a reduction in body weight associates with improved cardiovascular function. Interestingly, among treated mice, those with similar body weight to diabetic mice without treatment still seemed to have improved endothelial function (see Supplementary material online, *Tables S1* and S*2*), suggesting that the beneficial effects of rmSIRT1 on vascular function in large parts must be independent of the changes in body weight loss and plasma glucose levels.

Although the plasma level of SIRT1 remained below the detection level after chronic rmSIRT1, perhaps due to short half-life of SIRT1 in the circulation in mice, its concentrations were significantly increased in aortic tissue suggesting that circulating SIRT1 specifically targets the vasculature, although the mechanisms remain unknown yet. As non-histone proteins are frequently acetylated^42^ and as such post-translational modification is importantly involved in the signal transduction of membrane proteins,^43^ extracellular SIRT1 may regulate cellular function by cellular membrane deacetylation,^43,44^ and less likely by entering the cells due to its big molecular size.

Oxidative stress is critically involved in the pathogenesis of obesity and diabetes and particularly in vascular complications associated with these conditions.^45,46^ Here, we, for the first time, demonstrated that chronic treatment with rmSIRT1 improves endothelial and vascular responses of db/db mice with obesity-induced diabetes, at least partially by blunting oxidative stress. ROS such as superoxide anions are critical in scavenging and in turn inactivating endothelial-derived NO and enhancing endothelium-dependent contractions. Among other cellular sources, members of the NADPH oxidase (NOX) family are a major source of ROS and oxidative stress within the vasculature. NOX1, NOX2, NOX4, and NOX5 (the latter being absent in rodents) are the main NOX subtypes identified in vascular cells.^47^ The enhanced expression of each NOX isoform and the ensuing ROS formation have been directly associated with the severity of structural and functional alterations of the vascular wall in all major CVDs, including diabetes.^48,49^ While NOX expression differs in different vascular beds,^50^ NOX1 and 4 were up-regulated in aorta of diabetic mice. Upon chronic rmSIRT1 supplementation, NOX1 and NOX4, but not NOX2, were significantly down-regulated. Such a reduction of oxidative stress was further confirmed by a reduced protein carbonylation in aorta and total nitrotyrosine in plasma. Thus, improved vascular structure and function appears to be related to effects of rmSIRT1 on NADPH oxidase.

### 4.1 Study limitations

Certain limitations of our study warrant discussion. First, rmSIRT1 concentrations supplemented *in vivo* are based on cellular experiments (see Supplementary material online, *Figure S4*). Nonetheless, the dose chosen did reinstate vascular SIRT1 tissue levels to those of controls, suggesting proper dosing. Second, only male mice have been used, limiting the applicatility of our results to one sex. Other aspects related to vascular functions such as inflammation or lipids metabolism were not deeply investigated (Supplementary material online, *Figures S1 and S2*). Third, while the direct binding target of exogenous rmSIRT1 in vascular cells has to be identified by future studies, abundant SIRT1 protein were observed within vessels (Supplementary material online, *Figure S3*) and a marked interaction with endothelial cells was observed in the present study (see Supplementary material online, *Figure S5*). Fourth, as only mice with established diabetic phenotypes were studied, we can only report on the clinically potentially more relevant therapeutic but not preventive effects.

## 5. Conclusion

In summary, chronic supplementation of rmSIRT1 improves glucose metabolism and vascular function in mice with obesity-induced type 2 diabetes via reductions in NADPH-related oxidative stress, providing a potential therapeutic avenue for the treatment of diabetes-associated vascular dysfunction.

## Supplementary Material

cvad102_Supplementary_Data

## Data Availability

All data are incorporated into the article and its supplementary material.

## Notes

Translational perspective The growing burden of obesity and diabetes drives an increasing portion of atherosclerotic cardiovascular disease, and represents a major challenge to public health. Herein, we probe the efficacy of recombinant SIRT1 supplementation to preserve endothelial function and vascular compliance during diabetic conditions. Notably, SIRT1 levels were diminished in diabetic arteries of mice and humans alike, while recombinant SIRT1 delivery improved energy metabolism and vascular function by suppressing oxidative stress. Our study deepens mechanistic insights into the vasculo-protective effects conferred by recombinant SIRT1 supplementation and opens therapeutic avenues to mitigate vascular disease in diabetic patients.
