# Supplementary material for: Chronic SIRT1 supplementation in diabetic mice improves endothelial function by suppressing oxidative stress
Source: Cardiovasc Res. 2023 Jul 4;119(12):2190–201. doi: 10.1093/cvr/cvad102 (PMC10578911; doi:10.1093/cvr/cvad102)
Supplement: cvad102_Supplementary_Data [file cvad102_supplementary_data.docx]

Supplementary Material

**Chronic SIRT1 supplementation in diabetic mice improves endothelial function by suppressing oxidative stress**

**This file includes:**

Supplementary methods

Supplementary figures/tables Table 1 to Table 2 and S1 to S7

**Supplementary methods**

**RNA extraction and real-time quantitative polymerase chain reaction (qPCR)**

Frozen pieces of murine aortae were crushed mechanically with a mortar and pestle. Total RNA from crushed murine aortae or human endothelial cells (HAECs) was isolated with TRIzol (Invitrogen, Life Technologies Corporation, Zug, Switzerland) and reverse transcribed into cDNA by using High Capacity RNA-to-cDNA Kit (Applied Biosystems by Thermo Fisher Scientific, Zug, Switzerland) according to the manufacturer’s instructions. NanoDrop® ND1000 (NanoDrop Technologies, Wilmington, DE, USA) was used to assess concentration, purity, and integrity of isolated total RNA. Predesigned TaqMan Gene Expression Assays specific for murine *SIRT1* (Mm01168521_m1) and human *SIRT1* (HS01009006_m1) were used throughout, with (Mm99999915_g1) or human ACTB (Hs99999903_m1) serving as the reference genes. Respective controls (i.e., reverse transcription, genomic DNA contamination) were included in each run. Quantitative PCR was performed on a Quant Studio 5 cycler (Thermo Fischer Scientific, Zug, Switzerland) using a standard amplification protocol. Target gene mRNA expression levels were normalized to either *Gadph* or *ACTB* using the comparative CT method.

**Measurements of blood lipid and TNF-α**

Blood samples were obtained when mice were sacrificed, and blood lipid (Triglyceride, Cholesterol, HDL-c and LDL-c) were measured using commercial kits (EKF Diagnostics USA Stanbio Laboratory) according to manufactory’s instructions. The level of TNF- α was measured using Mouse TNF-alpha Quantikine HS ELISA Kit (R&D Systems).

**Immunohistochemical staining**

The aorta sample were fixed and processed as described in the main methods. The immunohistochemical staining of SIRT1 protein in the aorta tissue were performed by Sophistolab using anti-SIRT1 antibody (ab189494, Abcam, Cambridge, UK).

**Cell culture**

We cultured primary human aortic endothelial cells (HAEC) in complete endothelial cell medium (EGM™-2 Endothelial Cell Growth Medium-2, Lonza, Switzerland) in a humidified incubator with 5 % CO_2_ at 37 °C according to the supplier´s instructions. For experiments, 500,00 cells/well were seeded into 6-well plates for incubation with recombinant human SIRT1 protein (rhSIRT1) in dose response or effects of fructosamine after starvation for 24 hours under normal glucose or high glucose condition (HG, 25mmol/L D-glucose). Cells were harvested for protein detection by western blotting. For the observation of fluorescently tagged rhSIRT1 under microscope, HAECs were seeded on 4-well Nunc Lab-Tek Chamber Slide System (Permanox plastic, ThermoScientific) for incubation of tagged protein. After that, cells were washed with PBS for three times, followed by fixation with 4% paraformaldehyde for 10 minutes and then mounted with DAPI (ThermoScientific) for direct observation under microscope. SIRT1 protein was fluorescently labeled with ATTO-dye NHS-Esters (ATTO 488, ATTO-TEC GmbH, Germany) according to manufactory’s instructions.

**Supplementary Figures/Tables**

**Table 1:** Area under the curve (AUC) of the relaxations to acetylcholine (relax_AUC) in mice of different body weight (BW) suggesting that rmSIRT1 indeed seems to have additional effects on vascular function beyond the weight changes.

| db/db vehicle | | | db/db-rmSIRT1_high BW | | | db/db-rmSIRT1_low BW | | |
| --- | --- | --- | --- | --- | --- | --- | --- | --- |
|  | BW | relax_AUC |  | BW | relax_AUC |  | BW | relax_AUC |
| No.1 | 50.4 | 138.1 | NO.4 | 49.1 | 275.8 | No.7 | 33.6 | 296.6 |
| No.2 | 50.6 | 223.3 | NO.5 | 50.6 | 217.1 | No.8 | 38.7 | 292.1 |
| No.3 | 51.5 | 246 | NO.6 | 51.9 | 248.4 | No.9 | 43.4 | 236.5 |
| Mean | 50.8±0.6 | 202.5.2±56.5 | Mean | 50.5±1.4 | 247.1±29.3 | Mean | 38.6±4.9 | 275.1±33.5 |

**Table 2**. Glucose control and area under the curve (AUC) of the relaxations to acetylcholine (relax_AUC) in rmSIRT1-treated mice with different glucose levels suggesting that rmSIRT1 indeed seems to have effects on endothelial function independent of changes in glucose.

| db/db-rmSIRT1_high Glucose | | | db/db-rmSIRT1_low glucose | | |
| --- | --- | --- | --- | --- | --- |
|  | Glucose  (mmol/dL) | relax_AUC |  | Glucose (mmol/Dl) | relax_AUC |
| No.1 | 41.2 | 268.1 | NO.4 | 26.8 | 296.6 |
| No.2 | 38.8 | 236.5 | NO.5 | 29.4 | 195.1 |
| No.3 | 37.7 | 248.4 | NO.6 | 31.3 | 275.8 |
| mean±SD | 39.2±1.8 | 251±15.9 | mean±SD | 29.2±2.3 | 255.8±53.6 |
| ttest: high glucose v.s low glucose | *P*=0.004 | n.s. |  |  |  |


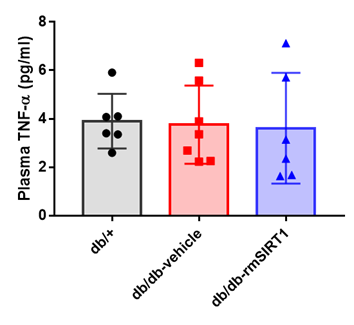


**Supplementary Fig. 1:** Plasma level of TNF-α in different of groups.

**
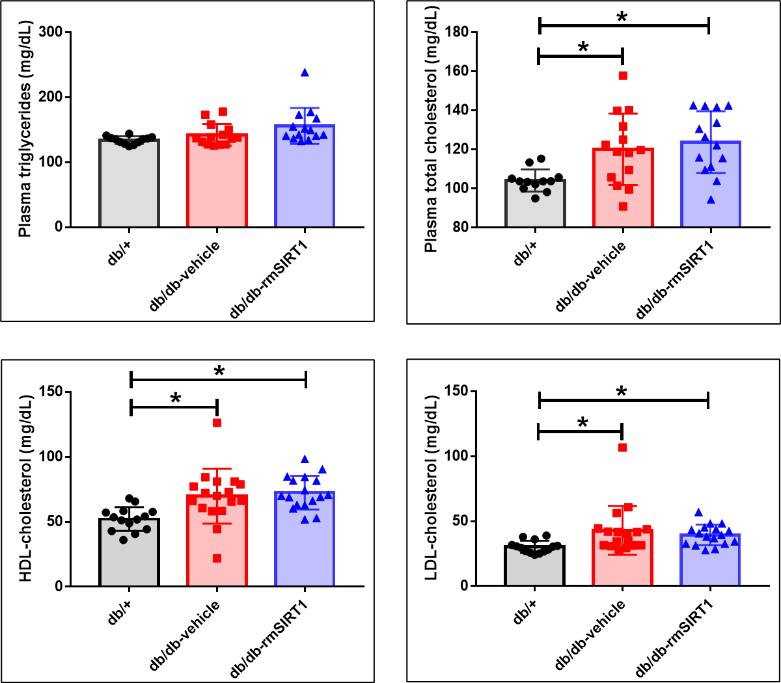
**

**Supplementary Fig. 2:** The level of triglycerides, total cholesterol, HDL-cholesterol and LDL-cholesterol in plasma from different groups of mice. Data are shown as mean ± SD. Data were analyzed using one-way ANOVA. *P<0.05.

**
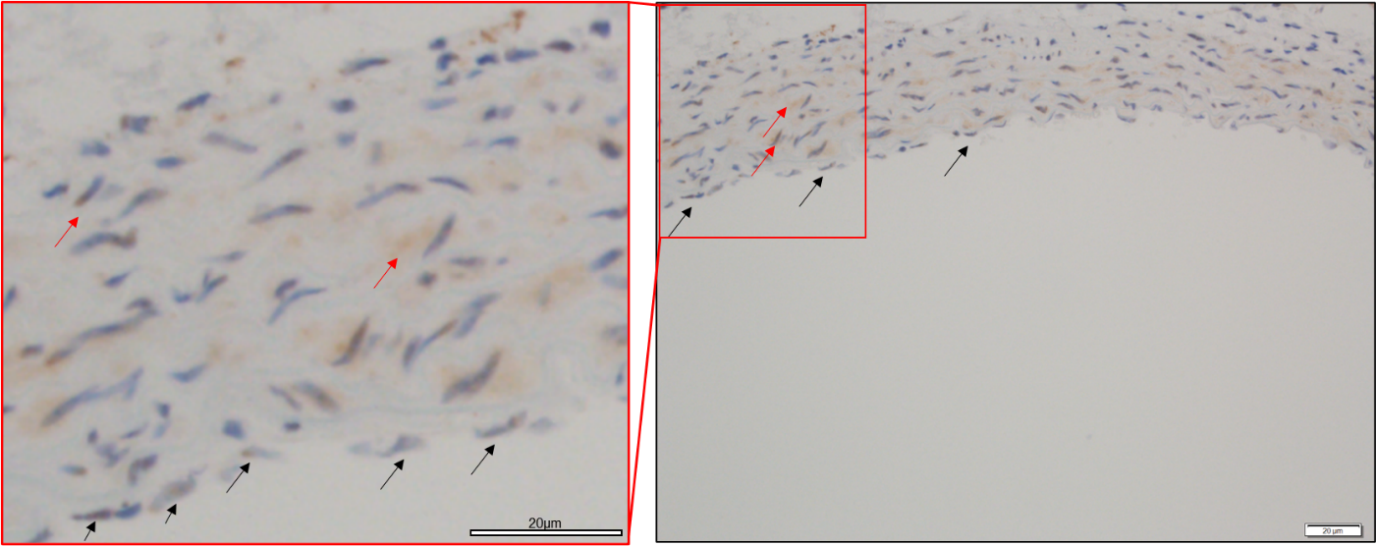
**

**Supplementary Fig. 3:** Immunohistostaining of SIRT1 protein aorta of mice. Red arrow indicates the signal in the media and black arrows indicate the localization of SIRT1 in the aortic intima.

**
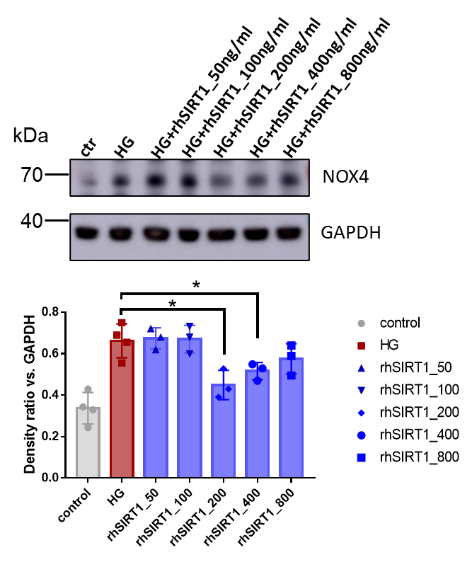
**

**Supplementary Fig. 4:**  Dose-response reaction of Human Aortic Endothelial Cells (HEACs) in expressing NOX4 with SIRT1 treatment. HEACs were subjected to high glucose for 48h comprising of 25mmol/L D-glucose with or without recombinant human SIRT1 protein (rhSIRT1) at concentrations of 50, 100, 200, 400, 800 ng/ml. *P<0.05 in comparison to HG condition.

Extension of the *in vitro* dose into mice:

Working concentration in HEACs: 200-400ng/ml, incubated for 48 hours

12-week-old db/db mice: ~50ml of body size

Dose: Working Concentration x body size =200ng/ml x 50ml = 10µg (per mouse)

In our animal studies, the mice were therefore injected 10µg of rmSIRT1 every two days i.p.


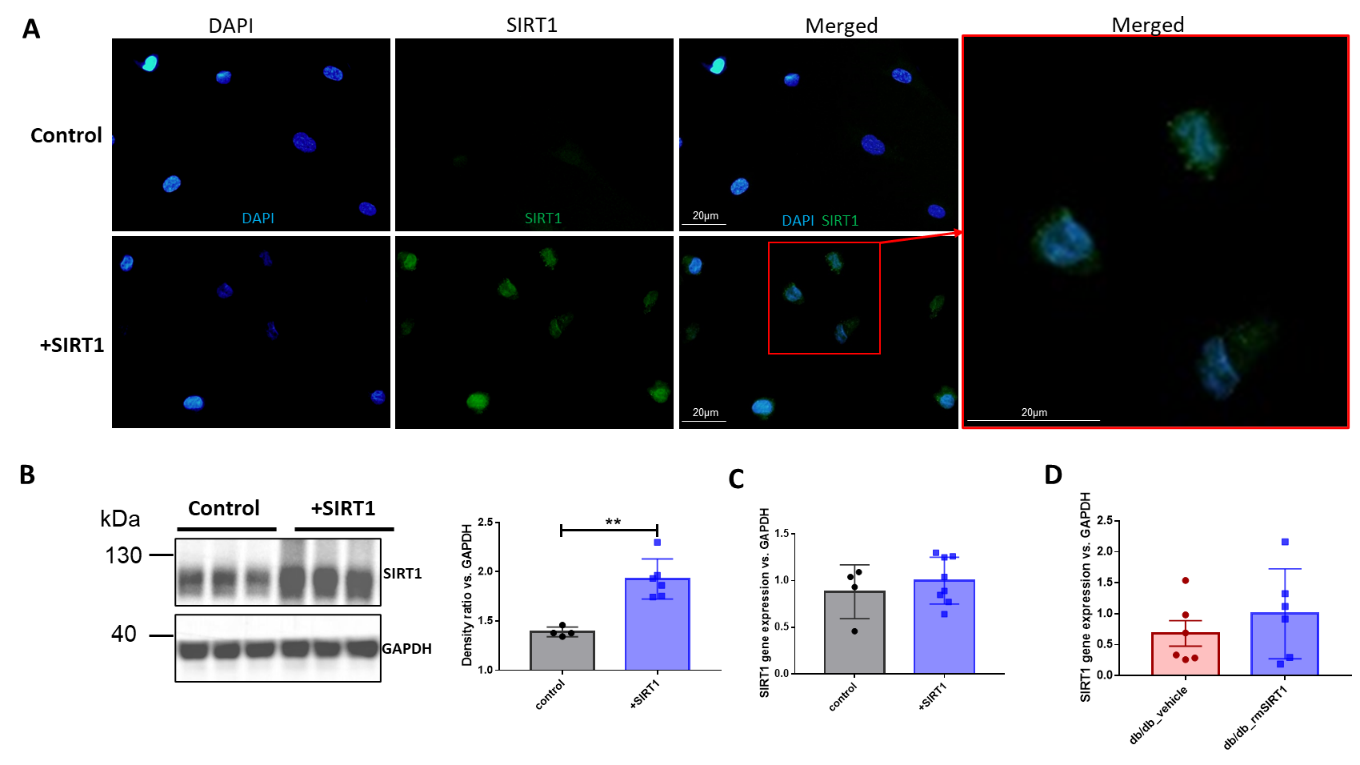


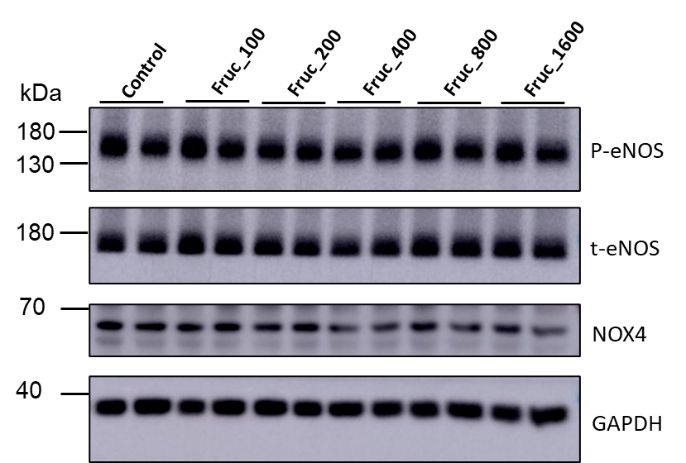
**Supplementary Fig. 5:** Protein and gene expression of SIRT1 in HAECs and in mouse aorta. (A) HEACs were incubated with Fluorescently tagged SIRT1 protein for 24 hours under HG condition and processed for microscopy analysis. (B) HEACs were incubated SIRT1 protein for 24 hours under HG condition and the protein expression of SIRT1 was assayed by Western blotting and (C) the gene expression by QPCR. (D) Gene expression of SIRT1 in aorta from db/db mice with or without rmSIRT supplementation. **P<0.01 in comparison. Student T test was used in comparisons.

**Supplementary Fig. 6:** Effects of fructosamine on endothelial function. HAECS were incubated with 100, 200, 400, 800 and 1600 µmol/L fructosamine for 24 hours. Levels of phosphorylated eNOS (S1177) and total eNOS were assayed by Western blotting using the cell lysates.


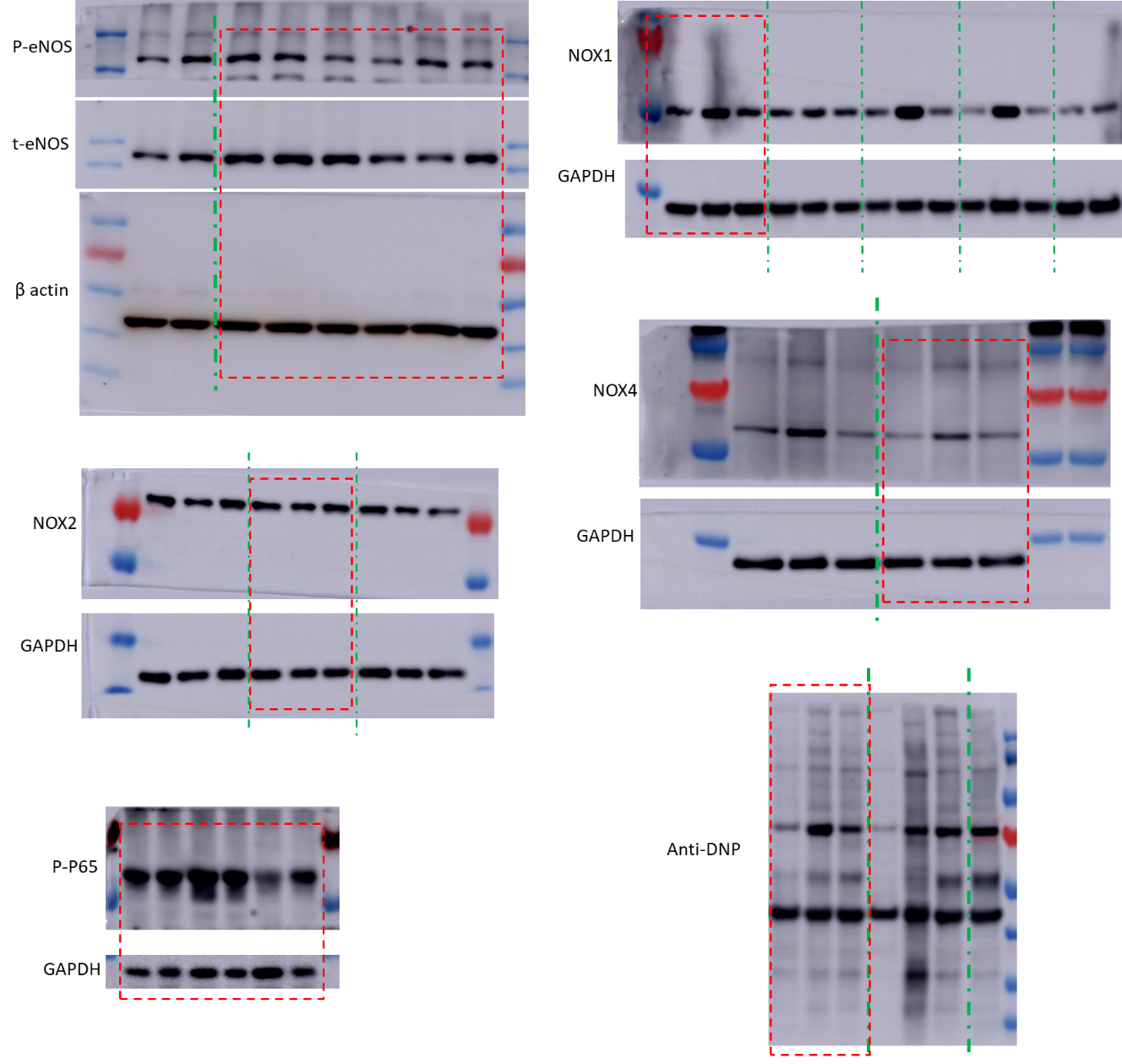


**Supplementary Fig. 7:** original images of the Western blotting gel. The cropped representative images used in the main figures are in the red rectangular area.
